# REAL-WORLD EXPERIENCE WITH JANUS KINASE INHIBITORS IN EXTRAINTESTINAL MANIFESTATIONS AND INFLAMMATORY BOWEL DISEASE IN COLOMBIA: A COMPARATIVE STUDY (JAKEIM-IBD STUDY)

**DOI:** 10.1590/S0004-2803.24612025-045

**Published:** 2026-01-09

**Authors:** Viviana PARRA-IZQUIERDO, Oscar ARDILA, Juan Ricardo MÁRQUEZ, Javier RIVEROS, Álvaro Andrés GÓMEZ-VENEGAS, Carlos Andrés MEDRANO-ALMANZA, Manuel BALLESTEROS, Jonathan BARRETO-PÉREZ, Juan Sebastian FRIAS-ORDOÑEZ

**Affiliations:** 1 Hospital Internacional de Colombia, Gastroenterology and Rheumatology, Bucaramanga, Colombia.; 2 El Bosque University, Cellular and Molecular Immunology Group- InMuBo, Bogota, Colombia.; 3 CES Clinic, Gastroenterology, Medellín, Colombia.; 4 Instituto ICO, Coloproctology, Medellin, Colombia.; 5 Clínica de La Mujer, Gastroenterology, Bogotá, Colombia.; 6 Private practice, Gastroenterology, Medellin, Colombia.; 7 Gastroadvanced IPS, Gastroenterology, Medellin, Colombia.; 8 Intergastro, Gastroenterology, Medellin, Colombia.; 9 Medicarte, Gastroenterology, Bogota, Colombia.; 10 Hospital Internacional de Colombia, Gastroenterology, Bucaramanga, Colombia.

**Keywords:** Extraintestinal manifestations, janus kinase inhibitors, inflammatory bowel diseases, therapy, therapeutics, disease management, drug-related side effects and adverse reactions, Manifestações extraintestinais, inibidores da janus quinase, doenças inflamatórias intestinais, terapia, terapêutica, manejo da doença, efeitos colaterais e reações adversas relacionadas a medicamentos

## Abstract

**Background:**

Extraintestinal manifestations (EIMs) significantly impact patients with inflammatory bowel disease (IBD). Janus kinase inhibitors (JAKi) are emerging as a potential treatment. This study describes real-world outcomes in Colombian patients with IBD and EIMs treated with upadacitinib or tofacitinib.

**Methods:**

Multicenter study including moderate-to-severe IBD patients with EIMs. We analyzed the prevalence, resolution, and progression of EIMs with JAKi therapy.

**Results:**

Among 77 patients (51 UC-tofacitinib, 16 UC-upadacitinib, 10 CD-upadacitinib), 28.6% (n=22) had EIMs, primarily articular (81.25%), followed by hepatobiliary (25%), cutaneous (18.75%), and ocular (6.25%). Most tofacitinib-UC patients (90%) had prior anti-TNF therapy. During induction, 66.6% had a clinical response, and 33.3% achieved EIM remission. In maintenance, 83.3% achieved remission. Among upadacitinib-UC patients, 66.6% had prior anti-TNF, 33.3% anti-integrin, and 33.3% were biologic-naïve. Corticosteroid use was reduced in 66.7%. Induction response was 66.7%, with 33.3% achieving remission. At six months, remission was 50%. No significant difference in remission rates was observed between upadacitinib and tofacitinib (OR 1.36, 95%CI 0.43-4.33, *P*=0.28). In CD-upadacitinib, all patients had prior anti-TNF therapy, with 66.6% achieving remission during induction. Adverse events included alopecia, acne, and herpes zoster.

**Conclusion:**

JAKi therapy is a safe and effective alternative for IBD patients with EIMs. While upadacitinib may offer superior intestinal benefits, both treatments demonstrated efficacy in managing EIMs.

## HIGHLIGHTS

•Upadacitinib and tofacitinib effectively controlled EIMs and reduced corticosteroid dependence.

•Safety profile was favorable, with mainly mild infections and herpes zoster.

•JAK inhibitors represent effective and safe options for IBD with extraintestinal involvement.

•Both agents achieved sustained remission of extraintestinal manifestations during maintenance therapy.

## RESUMO

**Contexto:**

As manifestações extraintestinais (MEIs) impactam significativamente os pacientes com doença inflamatória intestinal (DII). Os inibidores da Janus quinase (iJAK) estão surgindo como uma opção terapêutica promissora. Este estudo descreve os desfechos do mundo real em pacientes colombianos com DII e MEIs tratados com upadacitinibe ou tofacitinibe.

**Métodos:**

Estudo multicêntrico incluindo pacientes com DII moderada a grave e MEIs. Analisou-se a prevalência, resolução e progressão das MEIs com o tratamento com iJAK.

**Resultados:**

Entre 77 pacientes (51 com retocolite ulcerativa [RCU]-tofacitinibe, 16 RCU-upadacitinibe, 10 doença de Crohn [DC]-upadacitinibe), 28,6% (n=22) apresentaram MEIs, principalmente articulares (81,25%), seguidas por hepatobiliares (25%), cutâneas (18,75%) e oculares (6,25%). A maioria dos pacientes com RCU-tofacitinibe (90%) havia recebido terapia prévia com anti-TNF. Durante a indução, 66,6% apresentaram resposta clínica e 33,3% alcançaram remissão das MEIs. Na manutenção, 83,3% alcançaram remissão. Entre os pacientes com RCU-upadacitinibe, 66,6% haviam recebido anti-TNF, 33,3% anti-integrina e 33,3% eram biológicos naïve. O uso de corticosteroides foi reduzido em 66,7%. A resposta na indução foi de 66,7%, com remissão de MEIs em 33,3%. Aos seis meses, a remissão foi de 50%. Não houve diferença significativa nas taxas de remissão entre upadacitinibe e tofacitinibe (OR 1,36; IC95%: 0,43-4,33; *P*=0,28). Nos pacientes com DC-upadacitinibe, todos haviam recebido anti-TNF previamente, e 66,6% alcançaram remissão durante a indução. Os eventos adversos incluíram alopecia, acne e herpes-zóster.

**Conclusão:**

A terapia com iJAK é uma alternativa segura e eficaz para pacientes com DII e manifestações extraintestinais. Embora o upadacitinibe possa oferecer benefícios intestinais superiores, ambos os tratamentos demonstraram eficácia no manejo das MEIs.

## INTRODUCTION

Extra-intestinal manifestations (EIMs) in inflammatory bowel disease (IBD) are a significant challenge in its management due to their prevalence, impact on quality of life and treatment complexity. EIMs affect up to 50% of IBD patients and can involve multiple systems^1^ ^,^ ^2^. Their main impact is on quality of life and the clinical course of IBD. Some EIMs occur with active bowel disease, others independently^2^ ^,^ ^3^. This variability requires a nuanced approach, often involving interdisciplinary collaboration^4^. Treatment often involves biological therapies, particularly tumour necrosis factor (TNF) inhibitors such as infliximab and adalimumab. These have been shown to be effective in treating both IBD and associated EIMs, but EIMs can vary in their response to treatment^3^ ^,^ ^5^. Vedolizumab, an α4β7 integrin antagonist, has shown some promise in treating EIMs associated with luminal disease activity, but is less effective for EIMs that are independent of bowel inflammation^1^ ^,^ ^6^. Emerging therapies, including JAK inhibitors, interleukin-12/23 agents, and inhibitors interleukin 23 agents, are expanding the treatment landscape for EIMs, although their precise role and efficacy are still being investigated. Guidelines do not yet include specific treatment algorithms, complicating decision-making. This highlights the need for more robust clinical data and multidisciplinary approaches to manage EIMs in IBD^4^ ^,^ ^7^.

JAK inhibitors are an important option for treating IBD. They are also have potential benefits in treating other symptoms of IBD. The JAK-STAT pathway is critical for the signalling of several pro-inflammatory cytokines. These cytokines are involved in intestinal and extra-intestinal inflammation^8^ ^,^ ^9^. These include both selective and non-selective agents. Tofacitinib, a non-selective JAK inhibitor, is approved for moderate to severe UC and has shown efficacy in clinical trials^10^ ^,^ ^11^. However, there are safety concerns, including an increased risk of some diseases and major side effects. These require careful patient selection and monitoring, especially when considering long-term therapy^11^. JAK1 inhibitors, like upadacitinib and filgotinib, are effective against UC and are being investigated in CD^12^. These agents may be better for treating EIMs than non-selective JAK inhibitors because they may have a better safety profile, but long-term data are needed to confirm this^10^. Real-world data suggest upadacitinib is better than tofacitinib for ulcerative colitis (UC) after 12 months, but more research is needed on its effectiveness for extraintestinal manifestations (EIMs)^13^ ^-^ ^15^.

It is important to know how well JAK inhibitors work and if they are safe for patients with IBD and other health problems. JAK inhibitors like tofacitinib, upadacitinib and filgotinib are important options for treating IBD, especially UC and Crohn’s disease, because they can change several cytokine pathways involved in the development of these diseases^12^ ^,^ ^16^ ^-^ ^18^. JAK inhibitors are effective in inducing and maintaining remission in UC and CD. Clinical trials and meta-analyses show these agents induce remission in clinical, endoscopic and histological measures. They also offer rapid onset of action and oral administration, which are advantages over traditional biologics^16^ ^,^ ^19^ ^,^ ^20^. JAK inhibitors are a valuable option in refractory cases, even for patients who have failed conventional therapies^18^ ^,^ ^19^. Safety is a critical issue. JAK inhibitors have been associated with several adverse events, including an increased risk of infections and cardiovascular risks. The safety profile of different JAK inhibitors varies, with more selective JAK-1 inhibitors potentially offering a safer alternative^10^ ^,^ ^11^. Extraintestinal manifestations of IBD, are common and can significantly impact patients’ quality of life. JAK inhibitors may target these manifestations by targeting multiple cytokine pathways, although specific data on their efficacy in this context need further investigation^10^. There are limited specific studies from Latin America. A real-life Colombian study showed that tofacitinib is effective at resolving EIMs during induction in UC patients (75% saw improvement)^21^. This suggests that tofacitinib is an effective EIMs treatment for UC. Despite promising clinical trial data, there is a paucity of real-world evidence comparing the effectiveness of individual JAK inhibitors in managing extraintestinal manifestations (EIMs) in IBD^7^ ^,^ ^10^ ^,^ ^22^. While both tofacitinib and upadacitinib have demonstrated efficacy in moderate-to-severe UC and are under evaluation for CD, their comparative performance in controlling EIMs remains poorly defined. Notably, international guidelines offer limited direction on therapeutic selection for EIMs, particularly in biologic-experienced patients, and current recommendations are largely extrapolated from intestinal outcomes. Moreover, there is a lack of data from Latin American populations, who may present different clinical characteristics and access constraints. Therefore, this study aims to address this knowledge gap by evaluating and comparing the real-world outcomes of tofacitinib and upadacitinib in Colombian patients with IBD and active EIMs, contributing region-specific insights into their relative efficacy and safety.

## METHODS

### Study design

**FIGURE 1:**
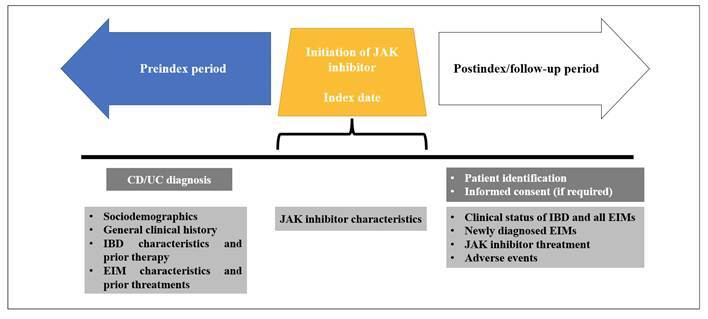
Study design.

This multicentre study was conducted from January 2022 to December 2024 at seven sites in Colombia. Patients with moderate to severe IBD and EIMs at the time of upadacitinib initiation (index date) with ≥3 months of follow-up after the index date, and patients with moderate to severe UC and EIMs at the time of tofacitinib initiation (index date) with ≥6 months of follow-up after the index date were included. Data collection spanned two periods: pre-index (from UC/CD diagnosis to 1 day before the index date) and post-index (from the index date to the date of chart abstraction, loss to follow-up, or death) (Figure 1). Retrospective data from the medical records of eligible IBD EIM patients who initiated upadacitinib or tofacitinib was abstracted into a web-based tool. Convenience sampling was used. Each centre had a different number of patients. The study was conducted according to the STROBE statement^23^.

CD: crohn’s disease. EIM: extraintestinal manifestation. IBD: inflammatory bowel disease. UC: ulcerative colitis.

### Study population

The study population consisted of patients aged 18 years and older with moderate-to-severe CD or UC with ≥1 active extraintestinal manifestation (EIM) within 8 weeks prior to starting upadacitinib or tofacitinib, which had not resolved at the time of starting the drug, these patients had to have at least 6 months of follow-up information after the starting date respectively. Patients were excluded if they had participated in an interventional clinical trial at the index date or during the follow-up period; had an unknown/unspecified type of IBD.

### Study outcomes

The main endpoint was the percentage of patients treated with upadacitinib or tofacitinib who had resolution of all EIMs within the induction phase^24^ ^,^ ^25^. As patients could have ≥1 EIM at the index visit, the primary outcome was resolution of all EIMs. Secondary outcomes were: (1) evolution and outcome of IEMs at 6 and 12 months after treatment; (3) new-onset EIMs during the post-index period. Newly diagnosed/de novo EIM or reactivated historical EIM during the post-index period (not active at the index date); upadacitinib/tofacitinib treatment persistence 6 months after initiation; persistence defined as ongoing treatment despite dose/schedule adjustments^24^ ^,^ ^26^ ^,^ ^27^.

In UC, clinical response was defined as a reduction in disease activity as measured by the adapted Mayo score, as a reduction of 3 or more Mayo clinical score points^28^. In CD, clinical response was defined as a reduction in stool frequency and abdominal pain by improving the frequency of very loose or liquid stools and the abdominal pain score, with an emphasis on the speed of symptom improvement in the induction phase of therapy, or a reduction in the CDAI score of more than 70 points^29^. In UC, clinical remission was defined as a Mayo total score ≤ 2, with no subscore > 1 and a rectal bleeding subscore of 0^28^. In CD, clinical remission was defined as a CDAI score of less than 150^29^.

### Concomitant therapy considerations

During the index treatment period, information was collected regarding concomitant use of immunosuppressive agents (e.g., azathioprine) or other biologics. Patients receiving overlapping therapy with anti-TNF, anti-integrin agents, or immunomodulators during the first 8 weeks of JAKi initiation were identified. In cases where prior biologics were tapered over a short period (<4 weeks) after JAKi initiation, this was considered a transition strategy rather than concurrent combination therapy. The potential impact of concomitant agents on EIM outcomes was documented and considered in the interpretation of treatment response. However, due to the retrospective design, no statistical adjustment for confounding effects was applied.

### Corticosteroid sparing definition and confounding considerations

Corticosteroid sparing was defined as either (1) complete discontinuation of systemic corticosteroids or (2) a documented reduction in dosage of ≥50% compared to the baseline dose at the start of JAK inhibitor therapy, as recorded in the medical records. Data on corticosteroid use were extracted at baseline, during induction, and at 6-month maintenance follow-up. The timing of corticosteroid tapering or discontinuation was also noted relative to EIM outcomes. Given that corticosteroids can impact both intestinal and extraintestinal inflammation, their use may have acted as a confounder in assessing EIM remission. To address this, remission outcomes were interpreted in the context of concurrent steroid exposure, and separate subgroup analyses of remission without corticosteroids were also considered.

### Assessment of baseline EIM severity

Given the retrospective nature of the study, formal grading of EIM severity at baseline was not systematically documented in the medical records. While some notes included qualitative descriptions (e.g., “severe axial pain”, “active PSC with cholestasis”, or “recurrent uveitis”), no standardized severity scale (e.g., BASDAI, dermatologic indices, or ophthalmologic scores) was used across centers. As such, remission was assessed as the resolution of symptoms or clinical findings regardless of initial severity, which may limit the interpretability of clinical impact.

### Definition and assessment of EIM response and remission

Due to the heterogeneity of EIMs in IBD, assessment of clinical response and remission was based on physician-reported outcomes documented in the medical records. A standardized definition across all types of EIMs is challenging given the differences in pathophysiology and clinical course. Therefore, remission of EIMs was defined pragmatically as complete resolution of symptoms and/or objective findings (e.g., normalization of laboratory values, disappearance of clinical signs, or imaging resolution), as recorded by the treating physician. Partial response was defined as noticeable improvement in symptoms or reduction in treatment needs without complete resolution. These assessments were based on expert clinical judgment, reflecting real-world practice, and were cross-validated when available with subspecialty notes (e.g., rheumatology, dermatology, hepatology). While not based on a unified scoring system due to retrospective data limitations, the use of medical chart documentation mirrors pragmatic approaches in routine clinical care and aligns with methodologies used in prior real-world EIM studies^22^ ^,^ ^26^.

### Interobserver variability and assessment consistency

Given the retrospective and multicenter nature of this study, no formal interobserver calibration or standardized training sessions were conducted prior to data abstraction. All outcome assessments-including EIM response and remission-were based on the documentation of the treating physician at each site. Although this may introduce variability in interpretation, it reflects real-world clinical practice in diverse care settings. To mitigate potential inconsistency, data abstraction was performed by experienced investigators at each center using a shared data collection tool and predefined operational definitions.

### Safety analysis

AEs were identified through retrospective review of clinical notes, laboratory results, and discharge summaries documented during the index treatment period. Due to the non-interventional nature of the study, no predefined AE monitoring protocol was implemented. However, sites adhered to routine clinical follow-up, including laboratory testing and clinical assessments every 8-12 weeks, per institutional practice. Adverse events were classified according to the Medical Dictionary for Regulatory Activities (MedDRA, version 20.0)^30^ and their severity was graded when possible using the Common Terminology Criteria for Adverse Events (CTCAE v5.0)^31^. Manage ment strategies, including drug discontinuation, dose adjustment, or supportive therapy (e.g., antiviral treatment), were recorded when explicitly mentioned in the medical records. Severe adverse events were defined as those requiring hospitalization, invasive intervention, or treatment interruption. 

### Statistical analysis

Data were processed using SPSS version 25.0. Categorical variables had percentages and 95% confidence intervals. Quantitative variables were described by mean, standard deviation, median and interquartile range. The chi-squared or Fisher’s exact test was used to compare categories, while the Mann-Whitney or t-test was used to compare quantitative variables, depending on the data distribution. A logistic regression model was used to identify variables associated with the likelihood of clinical response, remission and remission without corticosteroids during induction and maintenance. Odds ratios (OR) and 95% confidence intervals (95%CI) were reported. A ratio greater than 1 was a risk factor, a ratio less than 1 was a protective factor and a ratio of 1 was a neutral factor, according to the 95% confidence interval. The significance level for all tests was 0.05.

Comparative analyses were performed between treatment groups for baseline characteristics (Table 1) and types of EIMs (Table 2). For categorical variables, Chi-square or Fisher’s exact tests were used as appropriate. Continuous variables were compared using t-tests or Mann-Whitney U tests depending on normality assumptions and sample size. A two-sided *P*-value <0.05 was considered statistically significant. Given the small sample size, especially in subgroup comparisons, these results should be interpreted cautiously.

**TABLE 1:** Baseline and clinical characteristics of patients with inflammatory bowel disease (IBD) and extraintestinal manifestations (EIMs) treated with upadacitinib or tofacitinib.

Characteristic	UC-Upa (n=6)	CD-Upa (n=6)	UC-Tofa (n=10)	***P*(UC-Upa vs UC-Tofa)**	***P*(CD-Upa vs UC-Tofa)**	***P*(UC-Upa vs CD-Upa)**
Age, mean (SD) years	42.1(13.1)	41.5(12.6)	29.3(6)	0.0497	0.0602	0.8492
Female, n(%)	4(66.7)	3(50)	7(70)	1.0000	0.6066	1.0000
Weight, mean (SD) Kg	56(11.5)	50.3(9.9)	52.6(10.9)	0.2083	0.5938	0.0879
BMI, mean (SD) kg/m²	21.3(2.7)	19.7(7.4)	20.1(6.2)	0.6980	0.5690	0.3302
Smoking status, n(%)	2(33.3)	1(16.6)	2(10)	0.6044	1.0000	1.0000
Time since IBD diagnosis, mean (SD) years	8.9(7.3)	4.8(5.1)	7.6(4.9)	0.4134	0.8872	0.4348
Anti-TNF, n(%)	4(66.7)	6(100)	8(80)	0.6044	0.5000	0.4545
Corticosteroid, n(%)	4(66.7)	1(16.7)	9(90)	0.5179	**0.0076**	0.2424
Azathioprine, n(%)	4(66.7)	2(33.3)	2(20)	0.1181	0.6044	0.5671
Mesalazine, n(%)	6(100)	0(0)	6(100)	0.2335	**0.0338**	**0.0022**
Infliximab, n(%)	2(33.3)	5(83.3)	2(20)	0.6044	**0.0350**	0.2424
Adalimumab, n(%)	1(16.7)	4(66.7)	8(80)	**0.0350**	0.6044	0.2424
Golimumab, n(%)	1(16.7)	1(16.7)	0(0)	0.3750	0.3750	1.0000
Vedolizumab, n(%)	2(33.3)	5(83.3)	4(40)	1.0000	0.1451	0.2424

Comparative analysis of demographic and clinical variables across treatment groups. Continuous variables are presented as mean (standard deviation) and were compared using Student’s t-test or Mann-Whitney U test, depending on data distribution. Categorical variables are shown as absolute numbers and percentages and were compared using Fisher’s exact test. Statistically significant *P*-values (<0.05) are shown in bold. UC-Upa: patients with ulcerative colitis treated with Upadacitinib. CD-Upa: patients with Crohn’s disease treated with Upadacitinib. UC-Tofa: patients with ulcerative colitis treated with tofacitinib. SD: standard deviation. Anti-TNF: anti-tumor necrosis factor. BMI: body mass index.

### Ethical considerations

The Declaration of Helsinki, 2013 version, adopted in Fortaleza (Brazil)^32^, was used to inform the design, so that the research was considered risk-free and the confidentiality and integrity of the information collected were guaranteed. The study did not require submission to an Ethics Committee because it was a retrospective observational study based on the analysis of existing medical records. No experimental interventions, modifications to patient treatment, or prospective data collection were conducted. The study adhered to ethical guidelines by ensuring patient confidentiality and data anonymization, in compliance with local regulations and the Declaration of Helsinki. Since it involved de-identified real-world data without direct patient interaction, written informed consent was not required. Therefore, institutional ethical approval was deemed unnecessary under national and international ethical standards for non-interventional studies.

### Data availability statement

Data supporting the conclusions of this study are available on request from the corresponding author, VPI and JSFO. The data are not publicly available due to ethical or privacy restrictions, as they contain information that could compromise the privacy of research participants.

### Ethical statement

This research was reviewed and approved by the institutional review board and ethics committee of each institution. Its design took into account the requirements established in the Declaration of Helsinki, 2013 version, Fortaleza, Brazil, and it was considered a risk-free research. Confidentiality of the information collected from patients was guaranteed. The study did not require submission to an Ethics Committee because it was a retrospective observational study based on the analysis of existing medical records. No experimental interventions, modifications to patient treatment, or prospective data collection were conducted. The study adhered to ethical guidelines by ensuring patient confidentiality and data anonymization, in compliance with local regulations and the Declaration of Helsinki. Since it involved de-identified real-world data without direct patient interaction, written informed consent was not required. Therefore, institutional ethical approval was deemed unnecessary under national and international ethical standards for non-interventional studies.

## RESULTS

### Baseline characteristics

Of the 77 patients, 51 were on tofacitinib (UC) and 26 on upadacitinib (16 with UC and 10 with CD). 28.6% (n=22) had MEI (10 on tofacitinib) and 12 on upadacitinib (6 with UC and 6 with CD). The main characteristics of all patients are described in Table 1. Median age was 36.12 (range 18.5-68.7; SD 15.6) years, duration of IBD 7.2 (range 0.4-19.1; SD 5.7) years. In patients on Upadacitinib, the median time between diagnosis of IBD and initiation of therapy was 6.7 (range 0.25-18.6; SD 6.43) years. In patients on Tofacitinib, the median time between diagnosis of IBD and initiation of therapy was 4.5 (range 0.7-16.6; SD 5.1) years.

The most common nonbiological drug therapy was corticosteroids (63.6%, n=14), followed by mesalazine (54.5%%, n=11); and biological drug therapies included adalimumab (59.1%, n=13) and vedolizumab (50%, n=11) (Table 1). Among patients with EIMs, 4/22 (18.2%) continued on azathioprine during the induction phase, and 2 patients had overlapping therapy with anti-TNF agents for less than 4 weeks as part of a transition strategy. No patients remained on full dual therapy with biologics beyond 6 weeks. Table 2 shows the distribution of extraintestinal manifestations across the treatment groups. Articular EIMs (particularly arthritis and spondyloarthropathies) were the most frequently reported. No statistically significant differences were observed in the frequency of any specific EIM between groups. All patients in this study had ≥1 EIM, and the most common active EIMs reported at the index date (Table 2) were articular manifestations, including arthritis and, peripheral arthritis. No statistically significant differences were observed between groups in terms of baseline characteristics or EIM subtype distribution (*P*>0.05 for all comparisons), although descriptive trends were noted. 

**TABLE 2:** Distribution of extraintestinal manifestations (EIMs) by treatment group and disease subtype in patients with IBD (n=22).

EIM type	UC-Upa (n=6)	CD-Upa (n=6)	UC-Tofa (n=10)	***P*(UC-Upa vs UC-Tofa)**	***P*(CD-Upa vs UC-Tofa)**	***P*(UC-Upa vs CD-Upa)**
Autoimmune hepatitis	1(16.7)	0(0)	0(0)	0.3750	1.0000	1.0000
Primary sclerosing cholangitis	2(33.3)	0(0)	2(20)	0.6044	0.5000	0.4545
Ankylosing spondylitis	0(0)	1(16.7)	0(0)	1.0000	0.3750	1.0000
Arthralgias	1(16.7)	0(0)	1(10)	1.0000	1.0000	1.0000
Axial spondyloarthropathy	1(16.7)	0(0)	3(30)	1.0000	0.2500	1.0000
Arthritis	1(16.7)	2(33.3)	2(20)	1.0000	0.6044	1.0000
Peripheral arthritis	0(0)	2(33.3)	3 (30)	0.2500	1.0000	0.4545
Peripheral spondyloarthritis	1(16.7)	0(0)	2 (20)	1.0000	0.5000	1.0000
Psoriatic arthritis	0(0)	2(33.3)	0 (0)	1.0000	0.1250	0.4545
Erythema nodosum	0(0)	0(0)	2 (20)	0.5000	0.5000	1.0000
Psoriasis	0(0)	1(16.7)	0 (0)	1.0000	0.3750	1.0000
Scleritis	0(0)	1(16.7)	0 (0)	1.0000	0.3750	1.0000
Uveitis	0(0)	0(0)	1 (10)	1.0000	1.0000	1.0000
Aphthous oral ulcers	1(16.7)	0(0)	0 (0)	0.3750	1.0000	1.0000

Distribution of extraintestinal manifestations (EIMs) across treatment groups. Data are expressed as absolute numbers and percentages. Comparisons between groups were performed using Fisher’s exact test. No statistically significant differences were found. UC-Upa: ulcerative colitis treated with upadacitinib. CD-Upa: crohn’s disease treated with upadacitinib. UC-Tofa: ulcerative colitis treated with tofacitinib.

### Comparative analysis of baseline characteristics

A comparative analysis of baseline variables between treatment groups is presented in Table 1. Patients treated with UC-tofacitinib were significantly younger than those in the UC-upadacitinib group (*P*=0.0497). Statistically significant differences were also observed in prior corticosteroid use (CD-upadacitinib vs UC-tofacitinib, *P*=0.0076), mesalazine exposure (CD-upadacitinib vs UC-tofacitinib, *P*=0.0338; UC-upadacitinib vs CD-upadacitinib, *P*=0.0022), infliximab use (CD-upadacitinib vs UC-tofacitinib, *P*=0.0350), and adalimumab use (UC-upadacitinib vs UC-tofacitinib, *P*=0.0350). These differences likely reflect underlying disease subtype, prior treatment exposure, or therapeutic sequencing. No significant differences were found in BMI, smoking status, or time since IBD diagnosis.

### Study outcomes

**FIGURE 2:**
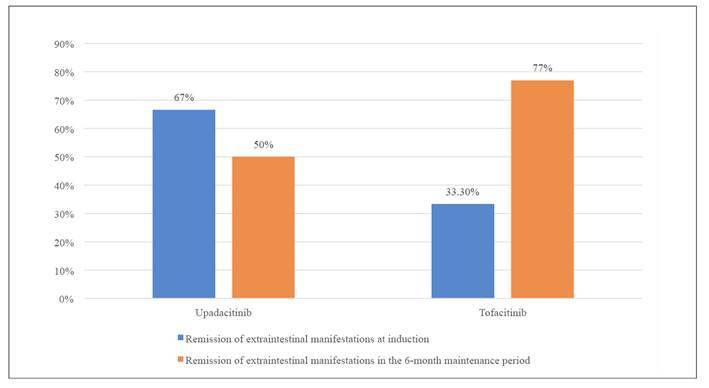
Remission of EIM according to JAK inhibitor agent during induction and maintenance periods in patients with UC and EIM.

Among tofacitinib-treated UC patients (n=10), during the induction phase, 50% (5/10) achieved remission of all EIMs, and 66.6% (4/6) showed a clinical response. In the maintenance phase, 40% (2/5) achieved remission, and 33.3% (1/3) had a clinical response. No new EIMs were observed post-index. For upadacitinib-treated UC patients (n=6), during induction, 33.3% (2/6) achieved remission of EIMs, and 66.7% (4/6) had a clinical response (Figure 2). In maintenance, 80% had been on treatment for 6 months, with 50% achieving remission of EIMs. Comparing the two treatments, there were no significant differences in the remission of EIMs between tofacitinib and upadacitinib after induction, with an odds ratio (OR) of 1.36 (95%CI 0.43-4.33; *P*=0.28). Both therapies had similar effects on extraintestinal manifestations and no new EIMs were observed during follow-up.

**No statistically significant differences were found. Remission of EIM at induction (*P*=0.152). Remission of extraintestinal manifestations in maintenance at 6 months (*P*=0.38).

Regarding ulcerative colitis (UC) disease activity, tofacitinib showed that 70% of patients achieved clinical remission during induction, and 30% had a clinical response. In the 6-month maintenance phase, 66.6% of patients had clinical remission, and 66.6% showed a clinical response. For upadacitinib, 33.3% (2/6) had clinical remission and 50% (2/4) had a clinical response during induction (FIGURE 2). In the 6-month maintenance phase, 66.7% achieved clinical remission and 66.7% had a clinical response. Upadacitinib demonstrated promising efficacy for UC, with improvements seen in both remission and response rates.

In Crohn’s disease (CD), among patients treated with upadacitinib, 33.3% achieved a clinical response, and 66.6% reached remission of EIMs during induction. During the maintenance phase, 83.3% achieved remission of EIMs. For IBD activity in CD, 16.7% (1/6) of patients achieved clinical remission at induction, and 80% (4/5) showed a clinical response, further highlighting the effectiveness of upadacitinib in managing Crohn’s disease alongside EIMs.

During induction, a significant proportion of patients treated with tofacitinib required corticosteroids, with 90% (9/10) needing steroids at the start of treatment. Among these patients, 30% (3/10) were able to reduce their steroid dose, and one patient experienced an increase in steroid dosage. By contrast, 40% (4/10) of patients continued to require corticosteroids during maintenance. In the upadacitinib-treated group, 66.7% (4/6) of UC patients required steroids prior to initiation, and 66.6% (2/3) of them successfully discontinued steroid use, with one patient achieving a dose reduction. No patients required the discontinuation of upadacitinib during the study. For CD patients on upadacitinib, 16.7% (1/6) required corticosteroids during induction, but the majority (83.3%) were able to reduce their steroid dose after induction. Both therapies demonstrated a capacity to reduce the reliance on corticosteroids, which is a key advantage in the management of IBD.

### Safety analysis

For tofacitinib patients (n=12), 30% (3/10) had infections without requiring in-hospital management. Also, alopecia areata and herpes zoster was observed in 1 patient. There were no cases of leukopenia, dyslipidaemia, cardiovascular events or thromboembolic events. In only one case was vaccination for Herpes zoster carried out, with the recombinant vaccine due to national availability.

Among patients with upadacitinib (n=12), in one case of a patient with CD, arthritis and scleritis, severe intrabadominal infection occurred, which required discontinuation of upadacitinib and surgical management for intrabadominal sepsis. There were no documented cases of leukopenia, worsening anaemia, hepatic or renal impairment. No cardiovascular or thromboembolic events were reported. 3 UC patients and 1 patient with CD were vaccinated for Herpes zoster. Acne and herpes zoster were also observed in 2 patients on upadacitinib. Patients with Upadacitinib had a lower risk of needing corticosteroid(OR 0.5. 95%CI 0.04-6.08; *P*=0.58).

## DISCUSSION

This study adds to the understanding of the role of JAK inhibitors in the management of extraintestinal manifestations in patients with inflammatory bowel disease. JAK inhibitors have been shown in trials to induce and maintain remission in UC and CD^12^ ^,^ ^15^ ^,^ ^16^ ^,^ ^18^ ^,^ ^19^. JAK inhibitors are effective in achieving clinical and endoscopic remission, with a rapid onset of action and a favourable safety profile^15^ ^,^ ^16^ ^,^ ^19^. However, there are concerns about infection and cardiovascular risks. The literature extensively covers the efficacy of JAK inhibitors in the intestinal manifestations of IBD. There is less detail, though, on their impact on extra-intestinal manifestations. JAK inhibitors modulate immune responses, so may affect EIMs like they do other autoimmune diseases^12^. Despite the methodological limitations and number of patients in the present study it presents valuable insights into the use of tofacitinib and upadacitinib forEIMs in patients with UC and CD. Both therapies have shown promising results, particularly in addressing EIMs, with no significant differences in their effectiveness. Tofacitinib-treated UC patients demonstrated substantial clinical response and remission in EIMs, with 50% achieving remission during induction and 66.6% showing a clinical response. In maintenance, a reduction in the presence of EIMs was observed, and no new EIMs emerged. Similarly, upadacitinib treatment led to notable improvements in EIMs, with 33.3% of UC patients achieving remission and 66.7% showing clinical response during induction, and 50% maintaining remission in the maintenance phase. This indicates that both treatments are effective at controlling EIMs in UC, without significant difference between them (OR 1.36, 95%CI 0.43-4.33; *P*=0.28).

Moreover, the requirement for corticosteroids was also an important factor in assessing the therapies’ overall effectiveness. A high percentage of tofacitinib-treated patients required steroids during induction, but several were able to reduce their steroid dose, and one patient even had an increase in the dose. In contrast, upadacitinib-treated patients showed greater success in reducing or discontinuing corticosteroids altogether, with 66.6% able to stop steroid use and only one patient requiring a dose reduction. The ability of both treatments to reduce or eliminate the need for corticosteroids is crucial, as it highlights the potential of these therapies in minimizing steroid dependence, a significant challenge in IBD management.

In CD, the results were consistent with previous findings for IBD therapies. A notable percentage of patients experienced clinical remission of EIMs during induction (66.6%), with a high rate of maintenance remission (83.3%). This supports the hypothesis that both tofacitinib and upadacitinib could be pivotal in controlling CD and associated EIMs, further extending their clinical value beyond the gut. The comparative effectiveness of tofacitinib and upadacitinib in the management of IBD has been explored in several studies^13^ ^-^ ^15^ ^,^ ^33^ ^-^ ^35^. One of the most outstanding is a real-world multicenter study in Japan also highlighted the higher efficacy of upadacitinib compared to tofacitinib, with upadacitinib-treated patients showing higher clinical remission rates and a lower risk of treatment discontinuation^15^. Regarding extraintestinal manifestations, while specific comparative data between tofacitinib and upadacitinib are limited, tofacitinib has been reported to benefit extraintestinal manifestations in IBD, particularly in UC, as per a literature review^35^. Upadacitinib has shown improvement in active immune-mediated diseases or extraintestinal manifestations in a real-world study, although specific comparative data with tofacitinib are not detailed^33^. In the present study, no differences were found between the two agents specifically for referral of extraintestinal manifestations.

In terms of safety, both tofacitinib and upadacitinib were generally well tolerated, with only a few adverse events observed. Among tofacitinib-treated patients, 30% (3/10) experienced infections, though none required hospitalization. Additionally, alopecia areata and herpes zoster were noted in one patient. Importantly, there were no serious adverse events such as leukopenia, dyslipidemia, cardiovascular complications, or thromboembolic events. However, only one patient received the Herpes zoster vaccine due to national vaccine availability. In the upadacitinib group, one patient with Crohn’s disease, arthritis, and scleritis experienced a severe intra-abdominal infection, which led to the discontinuation of upadacitinib and required surgical management. No serious issues such as leukopenia, anemia, hepatic, or renal dysfunctions were reported, and no cardiovascular or thromboembolic events occurred. Herpes zoster and acne were seen in two patients treated with upadacitinib. Notably, upadacitinib showed a lower risk of requiring corticosteroids compared to tofacitinib (OR 0.5; 95%CI 0.04-6.08; *P*=0.58), though this difference was not statistically significant. The study by Olivera et al. provides a comprehensive evaluation of the safety profile of Janus kinase (JAK) inhibitors, including tofacitinib and upadacitinib, in patients with inflammatory bowel diseases (IBD) and other immune-mediated diseases^11^. This systematic review and meta-analysis assessed the incidence rates of adverse events (AEs) and serious adverse events (SAEs) across a large cohort of patients. It found that while there is an increased risk of herpes zoster infection among patients treated with JAK inhibitors, other adverse events were not significantly increased compared to placebo or active comparators. This study is particularly valuable as it includes a broad range of data from randomized controlled trials and provides a detailed analysis of the safety concerns associated with JAK inhibitors in the context of IBD^11^. These study provides a robust foundation for understanding the safety profiles of tofacitinib and upadacitinib. No cardiovascular or thrombotic events were reported in our study as in other autoimmune pathologies such as rheumatoid arthritis^36^. Although the overall safety profile was acceptable, the retrospective design limited our ability to apply consistent monitoring and grading criteria across all centers. The lack of a uniform surveillance protocol may have led to underreporting of mild-to-moderate AEs. Nonetheless, severe adverse events were infrequent and clearly documented. Future prospective studies should incorporate standardized AE monitoring protocols and grading systems to better evaluate the safety of JAK inhibitors in patients with IBD and EIMs.

Vaccination against the herpes zoster virus is necessary for patients with inflammatory bowel disease (IBD) and extraintestinal manifestations who are about to start Janus kinase (JAK) inhibitor therapy because of the increased risk of herpes zoster reactivation. This risk is increased if they are treated with immunosuppressive therapies, including JAK inhibitors, which increase the incidence of herpes zoster infections^37^ ^,^ ^38^. The non-live recombinant zoster vaccine (RZV) is recommended for these patients as it provides effective protection against herpes zoster without the risks associated with live vaccines. The RZV has been shown to be immunogenic and safe in patients receiving JAK inhibitors, although the immune response may be reduced^39^ ^,^ ^40^. Therefore, the vaccine should be administered before starting JAK inhibitor therapy to maximise the protective effect^41^. Despite the limitations of the national healthcare system, our institutional group is trying to do this. Guidelines recommend the use of RZV in immunocompromised adults, including those on JAK inhibitors, to reduce the increased risk of herpes zoster. This approach is supported by evidence that the vaccine is well tolerated and effective in reducing the incidence of herpes zoster in this vulnerable population^38^ ^,^ ^40^.

This study has several important limitations. First, the small sample size-particularly in the ulcerative colitis groups treated with tofacitinib (n=10) and upadacitinib (n=6) limits the statistical power to detect meaningful differences and increases the risk of type II error. As such, the lack of statistically significant differences in EIM remission rates should be interpreted with caution, and these findings should not be extrapolated to inform clinical practice without further validation. Second, the retrospective design inherently limits data completeness and standardization. Clinical outcomes, including EIM response and remission, were based on physician judgment rather than validated instruments, and no formal interobserver calibration or centralized training was conducted across centers. Although operational definitions were applied during data abstraction, measurement bias and inter-site variability remain possible. Third, the severity of EIMs at baseline was not systematically assessed using validated scoring tools, making it difficult to fully contextualize the clinical relevance of observed remissions. Fourth, corticosteroid use during induction and maintenance phases may have acted as a confounding factor in EIM evaluation. While tapering or discontinuation was documented, the influence of steroids on inflammation cannot be fully disentangled in a retrospective framework. Fifth, a small subset of patients received concomitant immunomodulators or brief overlapping biologic therapies during treatment transition, which may have exerted synergistic effects on EIM outcomes. These potential confounders could not be statistically adjusted due to sample size limitations. Additionally, logistical barriers-such as fragmented outpatient care or limited access to follow-up laboratory testing in some centers-may have affected the completeness of monitoring data. Lastly, adverse event reporting relied on clinical documentation, and milder events may have been under-registered. Despite these limitations, the findings provide preliminary real-world evidence supporting the feasibility and potential effectiveness of JAK inhibitors in treating EIMs in IBD, and they highlight the urgent need for prospective, standardized studies in this area.

## CONCLUSIONS

In this multicenter retrospective study, both tofacitinib and upadacitinib showed potential clinical utility in the management of extraintestinal manifestations in patients with IBD. While favorable clinical responses and corticosteroid-sparing effects were observed, the findings must be interpreted with caution due to the limited sample size, retrospective design, and lack of standardized outcome assessment. These preliminary results support the rationale for further investigation of JAK inhibitors in this setting, and highlight the urgent need for prospective, controlled studies using validated outcome measures to confirm their safety and effectiveness in the treatment of EIMs.

## Data Availability

Not applicable - The study did not use research data
